# Facile fabrication of properties-controllable graphene sheet

**DOI:** 10.1038/srep24525

**Published:** 2016-04-15

**Authors:** Jin Sik Choi, Hongkyw Choi, Ki-Chul Kim, Hu Young Jeong, Young-Jun Yu, Jin Tae Kim, Jin-Soo Kim, Jin-Wook Shin, Hyunsu Cho, Choon-Gi Choi

**Affiliations:** 1 Creative Research Center for Graphene Electronics, Electronics and Telecommunications Research Institute (ETRI), 218 Gajeong-ro, Yuseong-gu, Daejeon 305-700, Korea; 2 Department of Advanced Chemical Engineering, Mokwon University, Daejeon 302-729, Korea; 3 UNIST Central Research Facilities (UCRF), Ulsan National Institute of Science and Technology (UNIST), Ulsan 689-798, Korea; 4 Division of Quantum Phases & Devices, Department of Physics, Konkuk University, Seoul 143-701 Korea; 5 Soft I/O interface Research Section, Electronics and Telecommunications Research Institute (ETRI), 218 Gajeong-ro, Yuseong-gu, Daejeon 305-700, Korea

## Abstract

Graphene has been received a considerable amount of attention as a transparent conducting electrode (TCE) which may be able to replace indium tin oxide (ITO) to overcome the significant weakness of the poor flexibility of ITO. Given that graphene is the thinnest 2-dimensional (2D) material known, it shows extremely high flexibility, and its lateral periodic honeycomb structure of sp^2^-bonded carbon atoms enables ~2.3% of incident light absorption per layer. However, there is a trade-off between the electrical resistance and the optical transmittance, and the fixed absorption rate in graphene limits is use when fabricating devices. Therefore, a more efficient method which continuously controls the optical and electrical properties of graphene is needed. Here, we introduce a method which controls the optical transmittance and the electrical resistance of graphene through various thicknesses of the top Cu layers with a Cu/Ni metal catalyst structure used to fabricate a planar mesh pattern of single and multi-layer graphene. We exhibit a continuous transmittance change from 85% (MLG) to 97.6% (SLG) at an incident light wavelength of 550 nm on graphene samples simultaneously grown in a CVD quartz tube. We also investigate the relationships between the sheet resistances.

Although graphene satisfies the transparency and flexibility requirements for transparent conducting electrode (TCE) applications, previously reported sheet resistance (Rs) values of CVD-grown single-layer graphene (SLG, ~ several hundred Ω/□)^1^,^2^,^3^,^4^,^5^,^6^,^7^,^8^,^9^,^10^,^11^,^12^ are significantly larger than that of indium tin oxide (ITO, ~ several tens Ω/□)^13^, which is widely used transparent conducting electrodes. In order to lower the Rs of graphene, chemical doping and layer-by-layer stacking of SLG transfer have been developed. Bae *et al.* achieved a remarkably low Rs of ~30 Ω/□ at ~90% transparency using both doping and layer-by-layer stacking methods^2^. However, the layer-by-layer stacking method requires repetitive transfer processes which includes the deposition of a supporting material, the etching of a metal catalyst, rinsing, and the elimination of the supporting material.

Another promising method is to control the number of layers of graphene by means of the segregation growth of multilayer graphene (MLG), as this method can facilitate the simple mass production of graphene at a low cost. Cu and Ni are well-known metal catalysts for the growth of large-area single- and multilayer CVD graphene, respectively^1^,^14^,^15^,^16^. Because Cu contains very few carbon atoms (<0.001 at.% at 1000 °C)^17^, graphene can be grown mostly on the Cu surface by the mechanism of chemisorption/deposition^14^,^15^, whereas relatively highly carbon-soluble Ni (~0.9 at.%)^15^ can be used to grow multilayer graphene from the surface and grain boundaries^15^,^16^. In addition, owing to the similar atomic characteristics of Cu and Ni, Cu-Ni alloys are easily formed through a high-temperature annealing process. Liu *et al.* reported graphene layer distributions while varying the thickness of the Ni layer of Cu/Ni structured film with respect to the atomic percentage of Ni in the Cu-Ni alloy^18^. Chen *et al.* exhibited thickness changes of grown graphene or graphite by varying certain aspects of the CVD growth condition, such as the deposition temperature and cooling rate while using Cu-Ni alloy foil^10^. However, these results show a lack of synthesis controllability, and their specific growth conditions are very sensitive and not compatible with the growth condition of Cu foil, widely used for the growth of single-layer graphene.

In this report, Cu and Ni are the only elements used as catalysts for synthesizing large-area graphene, and control of the optical and electrical properties of simultaneously grown multilayer and single-layer graphenes can be obtained under the same growth conditions for SLG growth with Cu foil. We demonstrate these controlled properties with two methods: graphene growth with Cu thickness control on a Cu/Ni metal catalyst structure, and the size-width control of a SLG-MLG mesh pattern using the optimized SLG growth condition derived from the first method.

## Results

**Figure 1:**
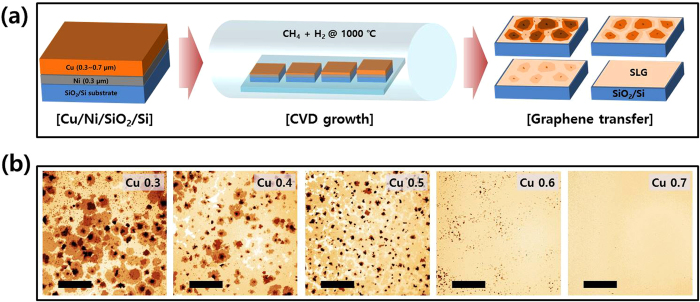
Graphene layer distributions grown on Cu thickness controlled Cu/Ni/SiO_2_/Si. (**a**) A schematics for preparing graphene samples using Cu/Ni metal catalyst structure. (**b**) Optical microscopic images of CVD graphenes transferred onto SiO_2_ substrate which simultaneously grown using Cu/Ni metal catalyst structure with varying the Cu thicknesses ranging from 0.3 to 0.7 μm, at fixed Ni thickness of 0.3 μm. Scale bar in (**b**) indicates 100 μm.

Figure 1 shows the preparation of our CVD graphene and the optical microscopy result of the CVD graphene transferred onto a SiO_2_ substrate. Our graphene samples were grown simultaneously on substrates structured with the Cu/Ni metal catalyst with various upper Cu thicknesses ranging from 0.3 μm to 0.7 μm in increments of 0.1 μm. The thickness of the Ni layer in Cu/Ni structure was fixed at 0.3 μm for all substrates. The same growth condition for single-layer graphene growth with the Cu foil was applied to our CVD growth condition (Supplementary Figure S1). After transferring onto SiO_2_ substrates, as shown in Fig. 1b, we discovered that the increase in the Cu thickness decreased the layer distribution until the Cu thickness reached 0.7 μm, at which uniform and continuous SLG without add-layers were noted. We were able to confirm that the add-layers, frequently observed in the Cu 0.6 μm condition, were significantly suppressed in our single-layer graphene grown in the Cu 0.7 μm condition (Supplementary Figure S2). In contrast, the layer number distributions increased as the Cu thickness decreased. For the Cu 0.6 μm case, few-layer graphene was limited close to the seed or the add-layer position, and the size and the number of the add-layers increased with a decrease in the Cu thickness from 0.6 μm to 0.3 μm.

As compared to a previous report on segregation graphene growth using CVD with the Cu/Ni metal catalyst structure^18^, our Cu/Ni structure also formed a uniform Cu-Ni alloy in terms of the depth via thermal annealing (at 1000 °C, 20 min) during the CVD graphene growth process (Supplementary Figure S3). The surface microstructure is changed during thermal annealing by the alloy and crystallization processes; however, the smooth morphology is maintained after graphene growth (Supplementary Figure S4). We managed to analyze the atomic ratios between Cu and Ni atoms through the depth profile at cross-sectional TEM measurements of the Cu 0.3 μm, Cu 0.5 μm and Cu 0.7 μm samples (Supplementary Figure S5). In our results, the atomic % of Ni after CVD growth corresponded to the thickness ratio of Ni to Cu and Ni, as shown in Table 1.

Through optical microscopy (Fig. 1b) and a TEM analysis (Table 1), few-layer graphene is considered to have formed from the central add-layer position, which appears to be extracted from the relatively highly carbon-soluble Ni layer. Moreover, the Cu/Ni thickness ratio in thermally formed Cu-Ni alloy may serve as a parameter to control the carbon solubility and thus determine the amount of carbon source necessary to grow graphene add-layers and lateral growth. Therefore, we were able to confirm that controlling the thickness of Cu in Cu/Ni is an effective means of controlling the graphene layer distributions.

**Figure 2:**
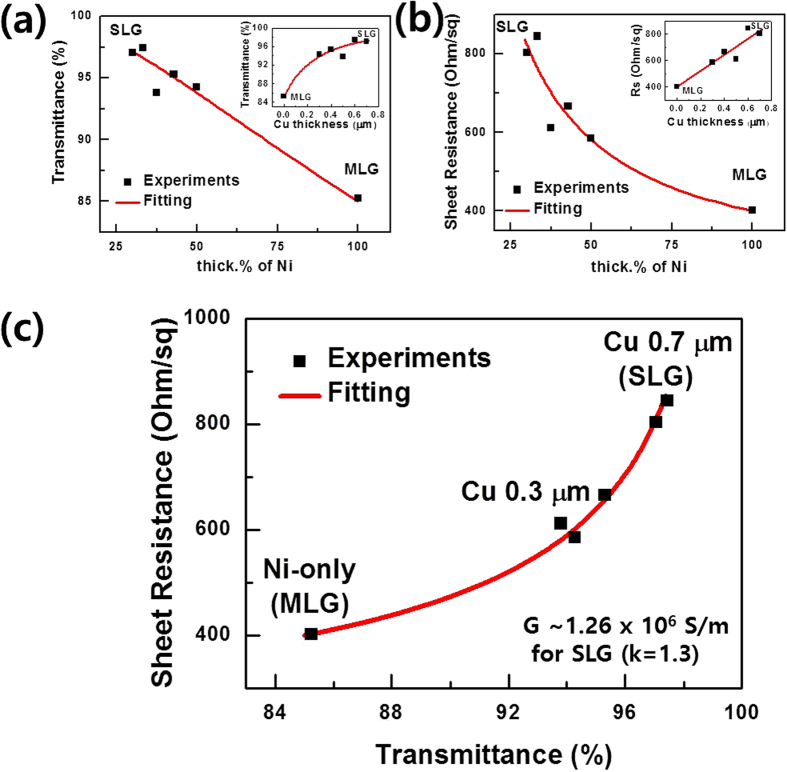
Thickness % of Ni dependent optical and electrical properties of graphenes transferred onto glass substrate. Ni thickness % dependent transmittance (at 550 nm) (**a**) and sheet resistance (**b**). Insets of (**a**,**b**) exhibit the Cu thickness dependences, respectively. (**c**) Plots of sheet resistance as a function of transmittance.

In order to confirm the optical and electrical properties of our layer-distribution-controlled graphene samples, we also transferred graphene samples onto glass substrates and then compared the optical Tr and electrical Rs characteristics as a function of the thickness % of Ni and the Cu thickness in Cu/Ni. Figure 2a,b show the changes in Tr and Rs depending on the calculated thickness % of Ni in the Cu/Ni structure. An Rs value of 804 Ω/□ at a Tr (at 550 nm) value of 97.05% were obtained for the Cu 0.7 μm sample (SLG), and the Ni-only (Cu 0 μm) sample exhibited an Rs value of 402 Ω/□ at a Tr value of 85.25% (~5–6 layers of graphene, MLG). Our Tr and Rs values for SLG are comparable to those in an earlier report by the authors of other SLG samples grown on Cu foil as well as other reported values of SLG samples which were transferred without a doping treatment^9^,^11^. Most interesting is that the thick.% of Ni vs the Tr plot (Fig. 2a) and the Cu thickness vs the Rs plot (Fig. 2b inset) show a linear relationship. If we adapt the linear relationship between Tr and the thick.% of Ni, the result is as follows,


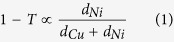


where 1-*T* denotes the light absorption rate at 550 nm, known to be 2.3% per graphene layer, and *d* is the film thickness. The light absorption is proportional to the thickness of the material; in this case, the average layer of the CVD-grown graphene (d_G,avg_) is proportional to the degree of light absorption and the thick.% of Ni. Moreover, because the areas of Cu and Ni are equal, the volume % of Ni is proportional to d_G,avg_. Thus, we assume that our method to control the Cu thickness of the Cu/Ni structure results in controlling the volume % of Ni, which then controls the amount of carbon supplied to the growing graphene and add-layers. Furthermore, the sheet resistance is inversely proportional to the film thickness as described by the following equation, 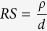, where *ρ* is the resistivity and *d* is the thickness. The other fittings (Fig. 2b; insets of Fig. 2a,b) are satisfied with a simple linear or inversely proportional function.

Figure 2c shows the relationship between Tr and Rs; and we confirm that the fitting follows the Beer-Lambert law^10^,^12^,


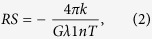


where *k* is the extinction coefficient, known to be 1.3 for graphene; *G* is the material conductivity; *λ* denotes the wavelength of the incident light (550 nm); and *T* is the transmittance of light. Using equation (2), the extracted *G* value is 1.26 × 10^6^ S/m. This value is comparable to the previously reported result of 1.1 × 10^6^ S/m from CVD-grown SLG^10^. In addition, we also find conditions exceeding the optimal Cu thickness for SLG growth (Cu 0.7 μm). When the Cu thickness increases beyond the optimal condition (Cu thickness ≥0.8 μm), cracks start to appear on the transferred graphene with the same tendency noted with the samples grown on pure Cu film^19^. We ascertained the effects of cracks on the properties of SLG in the optical microscopy images (to observe cracks) and via the Tr (>97.7%) and Rs values (≫1 kΩ/□) (Supplementary Figure S6).

**Figure 3:**
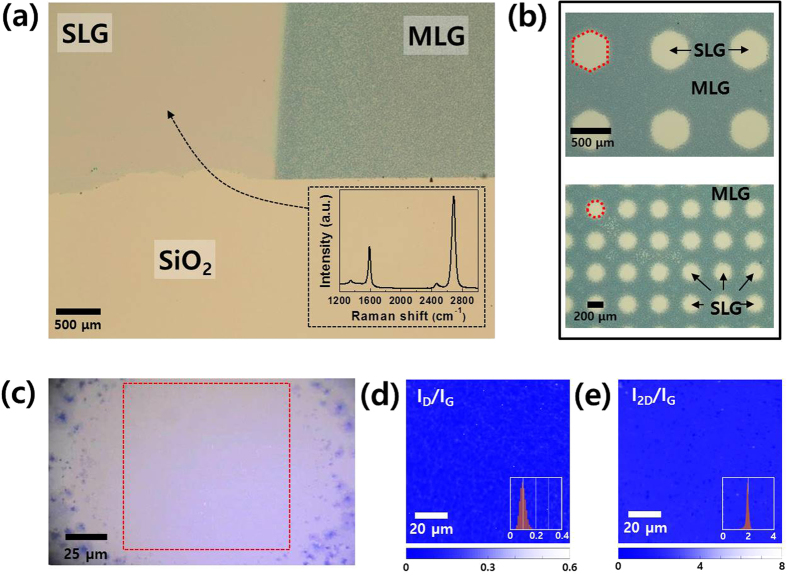
SLG-MLG patterned graphene transferred onto SiO_2_ substrate. (**a**) An optical microscopy image of SLG-MLG patterned graphene on SiO_2_ substrate. Inset shows a Raman spectrum obtained at SLG region. (**b**) Hexagon and circular shaped array pattern of SLG in MLG sheet. (**c**) Expanded optical microscopy image of a circular shaped SLG, and Raman mapping analysis of I_D_/I_G_ (**d**), and I_2D_/I_G_ (**e**) in the red-dashed square region designated in (**c**).

In order to determine the applicability of our Cu-thickness-controlled graphene, we deposited a 0.7-μm-thick Cu pattern using a shadow mask on a 0.3-μm-thick Ni-deposited SiO_2_ substrate (Ni/SiO_2_/Si). Figure 3a shows an optical microscopy image of the transferred result of the SLG-MLG pattern. We used the same CVD growth condition used in the previous experiments without patterns. The optical microscopy image of the transferred graphene onto the SiO_2_ substrate shows that the SLG-MLG patterns were continuously connected. During the thermal annealing process, Cu and Ni may diffuse vertically and laterally affect each other at the interface between the SLG and the MLG. However, the SLG area maintains an optimal condition without add-layers, despite the sensitive control over the Cu thickness, as shown in Fig. 1. Moreover, the Raman spectrum obtained at the SLG area exhibits the characteristics of the SLG with a small D peak and a single Lorentzian 2D peak, and the intensity ratio of the 2D and G peaks (I_2D_/I_G_) exceeds 2.

Furthermore, we succeeded in the fabrication of SLG array patterns (Fig. 3b). Although a shadow mask was used to deposit the Cu pattern so as to prevent the formation of defects such as the oxidation of metal catalysts or photoresist residue during the photolithography step, our transferred results show highly distinguishable SLG-MLG patterns with hexagon and circular shapes with dimeters exceeding 200 μm. We conducted AFM to compare the SLG and MLG topographies (Supplementary Figure S7) and analyzed the Raman characteristics of the patterned SLG region (200 μm diameter circle) via mapping. The Raman analysis results show uniform SLG characteristics with an I_D_/I_G_ ratio of approximately 0.1 and I_2D_/I_G_ ratio close to 2 with small distributions.

This SLG-MLG patterning growth enables the selective positioning of SLG elements for wafer-scale graphene device fabrication. One of the main topics in the research on graphene is large-area graphene growth and transfer processes^1^,^2^,^4^,^14^,^20^. A large uniform area of graphene is required for the mass production of graphene devices. However, nearly the entire area except for the graphene channel area (<several μm^2^) is etched away, followed by the formation of electrode patterns. If we use a MLG sheet with minimally patterned SLG at the graphene channel position, handling during the transfer process could be much easier than it is with large-area SLG-only graphene. Moreover, MLG can be utilized as an electrode^21^. We undertook 4-inch wafer-scale growth and transfer, finding that the optical and Raman characteristics in this case were uniform from the center to the side of the wafer-scale SLG-MLG sheet.

**Figure 4:**
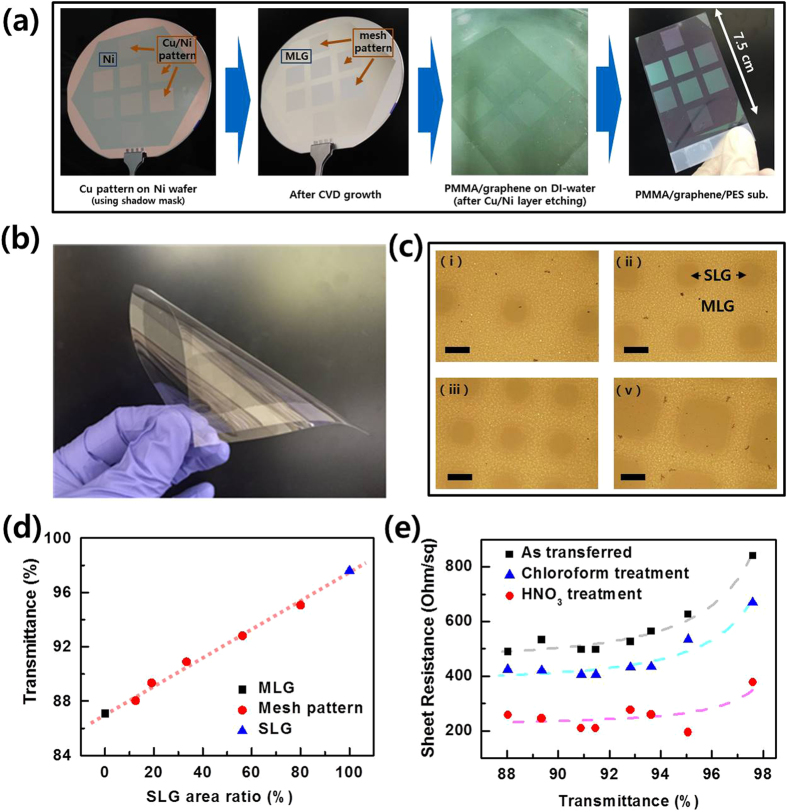
SLG-MLG mesh-patterns for flexible TCE. (**a**) A schematic of 4-inch wafer-scale SLG-MLG mesh-patterned graphene growth and transfer onto flexible substrate. (**b**) A display showing flexibility of transferred graphene sheet. (**c**) Optical microscopy images of size-width controlled SLG-MLG mesh patterns on glass substrate. (**d**) Plots of the transmittance as a function of SLG area ratio in SLG-MLG mesh-pattern. (**e**) Plots of the sheet resistance as a function of transmittance for SLG-MLG mesh-patterned sheet, and their chemical doping effects.

Although the fabrication of graphene-layer-controlled patterns for graphene-based devices is fascinating, forming various thickness-controlled Cu patterns on graphene sheets with various properties remains complicated. In order to achieve more efficient control of the optical and electrical properties of graphene such that these processes become applicable to TCE, we developed a mesh-patterned graphene sheet using only two optimal growth conditions of ‘Cu 0.7 μm/Ni 0.3 μm’ for SLG and ‘Ni 0.3 μm’ for MLG. Table 2 shows the details of the mesh pattern design, also showing the sizes of the square for SLG and the line widths for MLG. We designed 5 mesh patterns on a 4-inch substrate for wafer-scale growth and transfer. Figure 4a,b show the schematics and the resultant Cu 0.7 μm square pattern of SLG on a Ni/SiO_2_/Si substrate using a shadow mask. After CVD growth, we transferred the SLG-MLG mesh-patterned sheet onto flexible polyethersulfone (PES) film and a glass substrate with a supporting layer of PMMA. As shown in Fig. 4c, the SLG-MLG patterns are still distinguishable even after being transferred onto the transparent glass substrate in accordance with Cu patterns, as detailed in Table 2.

We analyzed the Tr value as a function of the SLG area ratio, finding a Rs value of 840 Ω/□ at a Tr (at 550 nm) value of 97.6% for the Cu 0.7 μm sample (SLG). The Ni-only (Cu 0 μm) sample exhibited an Rs value of 489 Ω/□ at a Tr value of 88% (~5–6 layers of graphene, MLG). Interestingly, the Tr results of the mesh-pattered films show linear variations between the MLG-only and the SLG-only films (Fig. 4d). We also observed that our mesh-patterned SLG-MLG sheet followed the Beer-Lambert law. For utilization of this SLG-MLG mesh sheet for TCE applications, a lower value of the sheet resistance is preferable to increase the performance of graphene-based devices by reducing the power consumption loaded at the electrode^22^. We carried out chemical doping with chloroform (CHCl_3_) and 63 wt% nitric acid (HNO_3_) for 5 minutes to reduce the sheet resistance. Chloroform has been used to reduce PMMA residue, but it is known to contribute to the doping effect of graphene^23^,^24^. In addition, 63 wt% HNO_3_ was reported as one of the most effective chemical agents for the p-doping of graphene^2^. As shown in Fig. 4e, the sheet resistance of the SLG-MLG mesh-patterned sheet is drastically reduced depending on the type of chemical used. The extracted material conductivity (G) of SLG is 1.42 × 10^6^ S/m, and the chloroform and HNO_3_ treatment resulted in values of 1.77 × 10^6^ S/m and 2.67 × 10^6^ S/m, respectively. Therefore, we were able to control the electrical properties of mesh-patterned graphene by means of chemical doping without changing the optical properties.

Previously, a heterostructure combining a mesh-type metal grid and graphene reportedly achieved a low Rs with high transparency for use as a TCE; however, the final product has a 3D structure^25^. In comparison, our mesh pattern has the form of planar SLG-MLG patterns, resulting in a 2D structure. Moreover, our SLG-MLG-mesh TCE sheet can be transferred to a flexible glass sheet and to, as already shown, SiO_2_, glass substrates, and transparent polymer substrates (Supplementary Figure S8). Furthermore, we assessed the applicability of our mesh-patterned graphene sheet as a TCE by fabricating an organic light-emitting diode (OLED) device (Supplementary Figure S9).

## Conclusion

In conclusion, here we introduced the optimal growth condition for single-layer graphene without add-layers by controlling the thickness of the Cu top layer in the Cu/Ni metal catalyst under the same growth condition used for single-layer graphene on Cu foil. Through the results, we discovered that both the electrical resistance and the optical transmittance could be controlled in simultaneously grown multilayer to single-layer graphene samples. Moreover, good control of the properties was achieved with a wafer-scale SLG-MLG mesh pattern. We therefore propose SLG-MLG-mesh patterned graphene as a 2D transparent conducting electrode given its, numerous potential applications in transparent and flexible devices. Moreover, the proposed fabrication technique, which offers control of the large-area thickness, could be beneficial for researchers of van der Waals heterostructures of the types which have gained attention recently.

## Methods

### Synthesis of Graphene

The graphene was synthesized on Cu/Ni metal catalyst layers by the thermal chemical vapor deposition (T-CVD). For Cu (0.3 ~ 0.7 μm) and Ni (0.3 μm) metal catalysts, DC sputter and E-beam evaporator were used to deposit on a thermally oxidized SiO_2_ substrate (300 nm), respectively. The Cu/Ni/SiO_2_/Si substrate was heated up to 1,000 °C inside of a quartz tube under H_2_ atmosphere, and then the graphene was grown on the Cu/Ni/SiO_2_/Si substrate flowing gas mixtures of H_2_ : CH_4_ = 10 : 15 (sccm) for 20 min. After growing the graphene, PMMA (950 PMMA A6), which served as an adhesive supporting layer, was spin-coated on a graphene at 3000 RPM for 30 s. The PMMA/graphene/metal catalyst was separated from Si substrate during floating on buffered HF (BOE). It takes several minutes for separation. Then, the PMMA/graphene/metal catalyst was floated on 0.1 M ammonium persulfate solution (APS) to etch the metal catalyst. After rinsed the PMMA/graphene in DI-water over a few hours, the PMMA/graphene was transferred to the target substrate and baked at 75 °C for few minutes for increasing the adhesion between graphene and target substrate. The PMMA was removed with acetone and IPA.

### Transmittance (Tr) and sheet resistance (Rs) analysis

The optical transmittance was obtained using S-4100 PDA UV-Vis spectrophotometer from SCINCO. The sheet resistance was measured based on the van der Pauw method by using HL5500IU Hall system from ACCENT. In order to carry out the optical property measurement, the graphene sample was transferred onto transparent substrate. Indium ingot (99.999%, Lot #C17 × 050, Alfa Aesar) was used as a contact pad for measuring sheet resistance by attaching on 4 edges of rectangular shape transferred graphene.

## Additional Information

**How to cite this article**: Choi, J. S. *et al.* Facile fabrication of properties-controllable graphene sheet. *Sci. Rep.* **6**, 24525; doi: 10.1038/srep24525 (2016).

## Supplementary Material

Supplementary Information

## Figures and Tables

**Table 1:** Prepared Cu thicknesses on Ni (0.3 μm) structure, calculated thickness % of Ni, and atomic % of Ni through measuring cross-sectional TEM after CVD growth.

Cu thick. in Cu/Ni	0.3 μm	0.4 μm	0.5 μm	0.6 μm	0.7 μm
thick.% of Ni (cal)	50%	~43%	~37%	~34%	30%
at.% of Ni (TEM)	49.47%		39.44%		30.79%

**Table 2:** Square size and line width of Cu pattern on Ni film for fabricating SLG-MLG mesh-patterned TCE, and their calculated SLG area ratio, and the measured transmittance results.

Pattern #	square size (μm)	Line width (μm)	SLG area ratio (%)	Transmittance (%)
(i)	100	200	12.5	88.04
(ii)	100	150	19.05	89.35
(iii)	100	100	33.33	90.9
(iv)	150	100	56.25	92.82
(v)	200	100	80	95.07

## References

[b1] Kim K. S. *et al.* Large-scale pattern growth of graphene films for stretchable transparent electrodes. Nature 457, 706–710 (2009). 19145232 10.1038/nature07719

[b2] Bae S. *et al.* Roll-to-roll production of 30-inch graphene films for transparent electrodes. Nature Nanotech. 5, 574–578 (2010). 10.1038/nnano.2010.132 20562870

[b3] Chen Z., Cotterell B. & Wang W. The fracture of brittle thin films on compliant substrates in flexible displays. Eng. Fract. Mech. 69, 597–603 (2002).

[b4] Ryu J. *et al.* Fast synthesis of high-performance graphene films by hydrogen-free rapid thermal chemical vapor deposition. ACS Nano 1, 950–956 (2014). 24358985 10.1021/nn405754d

[b5] Geim A. K. & Novoselov K. S. The rise of graphene. Nature Mater. 6, 183–191 (2007). 17330084 10.1038/nmat1849

[b6] Nair R. R. *et al.* Fine structure constant defines visual transparency of graphene. Science 320, 1308 (2008). 18388259 10.1126/science.1156965

[b7] Muñoz R. & Gómez-Aleixandre C. Review of CVD synthesis of graphene. Chem. Vap. Deposition 19, 297–322 (2013).

[b8] Seah C. -M., Chai S. -P. & Mohamed A. R. Mechanisms of graphene growth by chemical vapour deposition on transition metals. Carbon 70, 1–21 (2014)

[b9] Suk J. W. *et al.* Transfer of CVD-grown monolayer graphene onto arbitrary substrates. ACS Nano 5, 6916–6924 (2011). 21894965 10.1021/nn201207c

[b10] Chen S. Synthesis and characterization of large-area graphene and graphite films on commercial Cu-Ni alloy foils. Nano Lett. 11, 3519–3525 (2011). 21793495 10.1021/nl201699j

[b11] Reina A. *et al.* Large area, few-layer graphene films on arbitrary substrates by chemical vapor deposition. Nano Lett. 9, 30–35 (2009) 19046078 10.1021/nl801827v

[b12] Cai W., Zhu Y., Li X., Piner R. D. & Ruoff R. S. Large area few-layer graphene/graphite films as transparent thin conducting electrodes. Appl. Phys. Lett. 95, 123115 (2009).

[b13] Granqvist C. C. & Hultåker A. Transparent and conducting ITO films: new developments and applications. Thin Solid Films 411, 1 (2002).

[b14] Li X. *et al.* Large-area synthesis of high-quality and uniform graphene films on copper foils. Science 324, 1312–1314 (2009). 19423775 10.1126/science.1171245

[b15] Li X., Cai W., Colombo L. & Ruoff R. S. Evolution of graphene growth on Ni and Cu by carbon isotope labeling. Nano Lett. 9, 4268–4272 (2009). 19711970 10.1021/nl902515k

[b16] Zhang Y. *et al.* Comparison of graphene growth on single-crystalline and polycrystalline Ni by chemical vapor deposition. J. Phys. Chem. Lett. 1, 3101–3107 (2010).

[b17] López G. A. & Mittemeijer E. J. The solubility of C in solid Cu. Scr. Mater. 51, 1–5 (2004).

[b18] Liu X. *et al.* Segregation growth of graphene on Cu-Ni alloy for precise layer control. J. Phys. Chem. C 115, 11976–11982 (2011).

[b19] Tian J. *et al.* Surface structure deduced differences of copper foil and film for graphene CVD growth. Appl. Surf. Sci. 300, 73–79 (2014).

[b20] Lee J. -H. *et al.* Wafer-scale growth of single-crystal monolayer graphene on reusable hydrogen-terminated germanium. Science 344, 286–289 (2014). 24700471 10.1126/science.1252268

[b21] Park J. -U., Nam S., Lee M. -S. & Lieber C. M. Synthesis of monolithic graphene-graphite integrated electronics. Nature Mater. 11, 120–125 (2012). 22101813 10.1038/nmat3169 PMC3602909

[b22] Kang J. *et al.* High-performance graphene-based transparent flexible heaters. Nano Lett. 11, 5154–5158 (2011). 22082041 10.1021/nl202311v

[b23] Kim H. H. *et al.* Substrate-induced solvent intercalation for stable graphene doping. ACS Nano 7, 1155–1162 (2013). 23368414 10.1021/nn306012p

[b24] Wood J. D. *et al.* Annealing free, clean graphene transfer using alternative polymer scaffolds. Nanotechnology 26, 055302 (2015). 25580991 10.1088/0957-4484/26/5/055302

[b25] Zhu Y., Sun Z., Yan Z., Jin Z. & Tour J. M. Rational design of hybrid graphene films for high-performance transparent electrodes. ACS Nano 5, 6472–6479 (2011). 21774533 10.1021/nn201696g

