# Supplementary material for: Facile fabrication of properties-controllable graphene sheet
Source: Sci Rep. 2016 Apr 15;6:24525. doi: 10.1038/srep24525 (PMC4832197; doi:10.1038/srep24525)
Supplement: Supplementary Information [file srep24525-s1.doc]

**SUPPLEMENTARY INFORMATION**

**Facile fabrication of properties-controllable graphene sheet**

**Jin Sik Choi^1+^, Hongkyw Choi^1+^, Ki-Chul Kim^2^, Hu Young Jeong^3^, Young-Jun Yu^1^, Jin Tae Kim^1^, Jin-Soo Kim^4^,** Jin-Wook Shin^5^, Hyunsu Cho^5^, **and Choon-Gi Choi^1*^**

^1^ Creative Research Center for Graphene Electronics, Electronics and Telecommunications Research Institute (ETRI), 218 Gajeong-ro, Yuseong-gu, Daejeon 305-700, Korea

^2^Department of Advanced Chemical Engineering, Mokwon University, Daejeon 302-729, Korea

^3^UNIST Central Research Facilities (UCRF), Ulsan National Institute of Science and Technology (UNIST), Ulsan 689-798, Korea

^4^Division of Quantum Phases & Devices, Department of Physics, Konkuk University, Seoul 143-701 Korea

^5^ Soft I/O interface Research Section, Electronics and Telecommunications Research Institute (ETRI), 218 Gajeong-ro, Yuseong-gu, Daejeon 305-700, Korea

**^+^**These authors contributed equally to this work.

^*^email: cgchoi@etri.re.kr


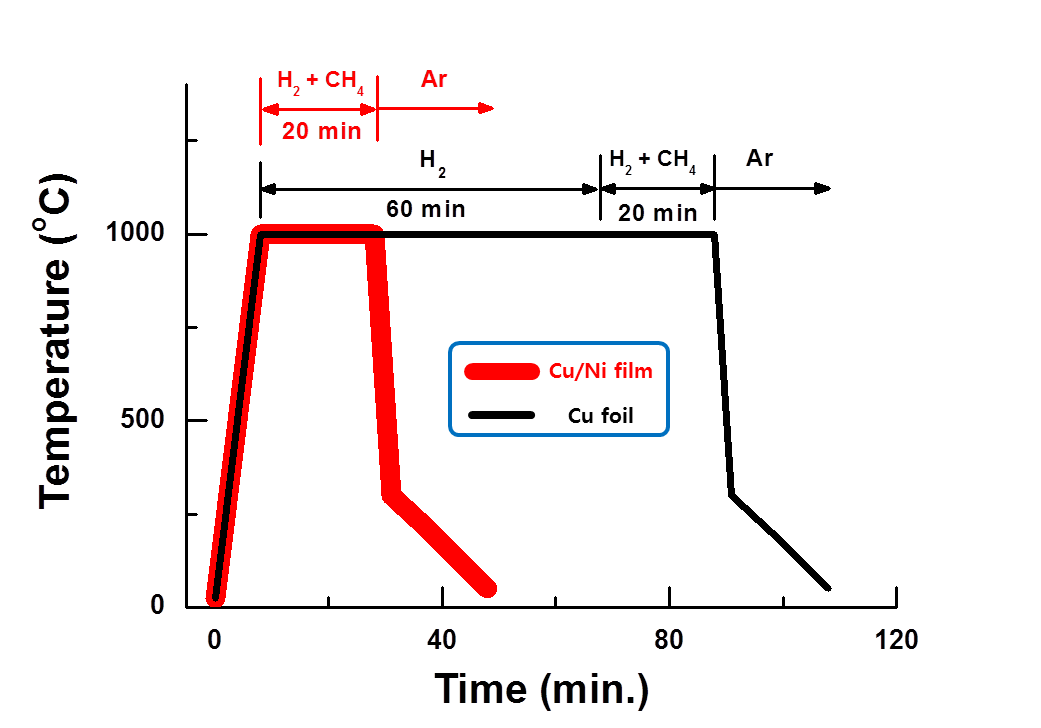


**Figure S1. Graphene growth conditions of Cu/Ni film vs Cu foil.** 2" tube-type quartz furnace was used with movable ramp heater between growth zone and heating/cooling zone for rapid heating and cooling. The time-dependent temperature changes at the growth zone are exhibited with their inlet gas information. The same mixture of gases of H_2_ and CH_4_ with ratio of 10:15 sccm for 20 minutes were used for growing graphene. A difference between Cu/Ni film and Cu foil is conducting an annealing process before graphene growth. The pre-annealing has been conducted to grow the Cu grain size of Cu foil for obtaining high quality graphene^S1,S2^, however the effect was not crucial in case of the Cu/Ni film.


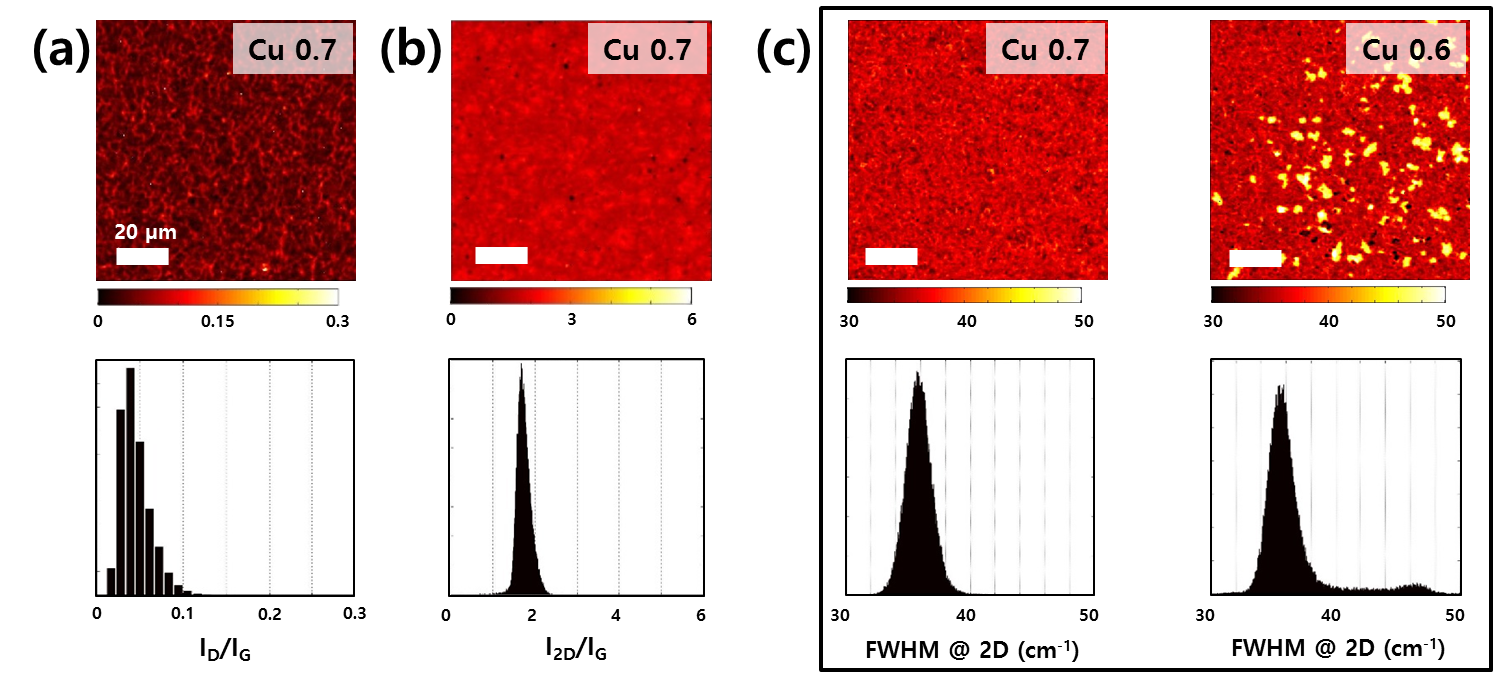


**Figure S2. Raman analysis of SLG obtained from Cu/Ni metal catalyst.** The Raman mapping was obtained in 100 x 100 μm^2^ area with 512 x 512 pixel information. Intensity ratio of D & G peaks (I_D_/I_G_, **a**), and 2D & G peaks (I_2D_/I_G_, **b**) were analyzed. **c,** exhibits an analysis of full with at half maximum (FWHM) of 2D peaks for add layer observation^S3^ with comparing graphenes grown on 'Cu0.7/Ni0.3' and 'Cu0.6/Ni0.3'.


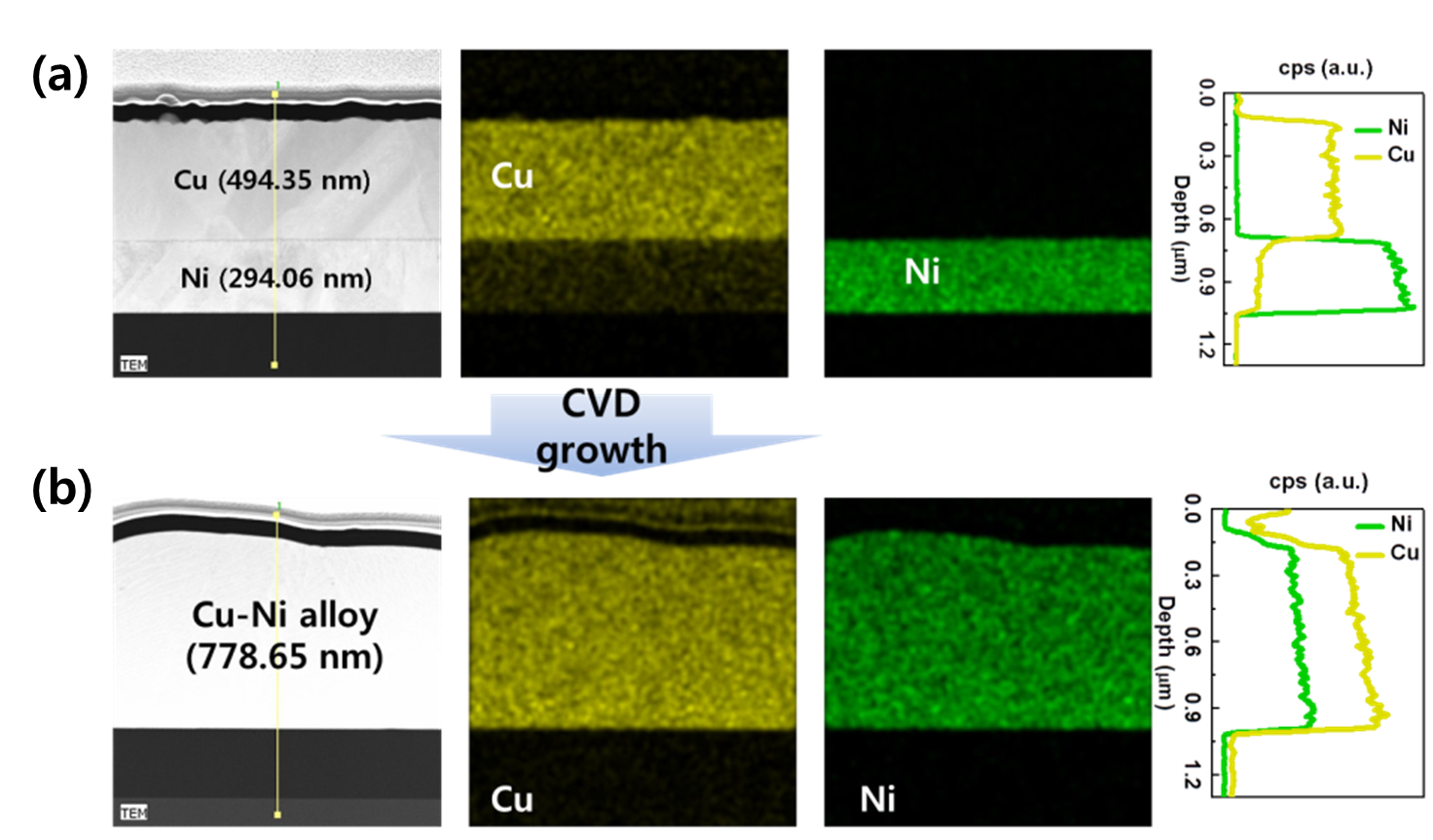


**Figure S3. Uniform Cu-Ni alloy formation during CVD growth.** Cross-sectional HRTEM (high resolution transmission electron microscopy) and EDS (energy disperse X-ray spectrometry) analysis on ‘Cu0.5/Ni0.3’ film that comparing before (**a**) and after (**b**) CVD growth.


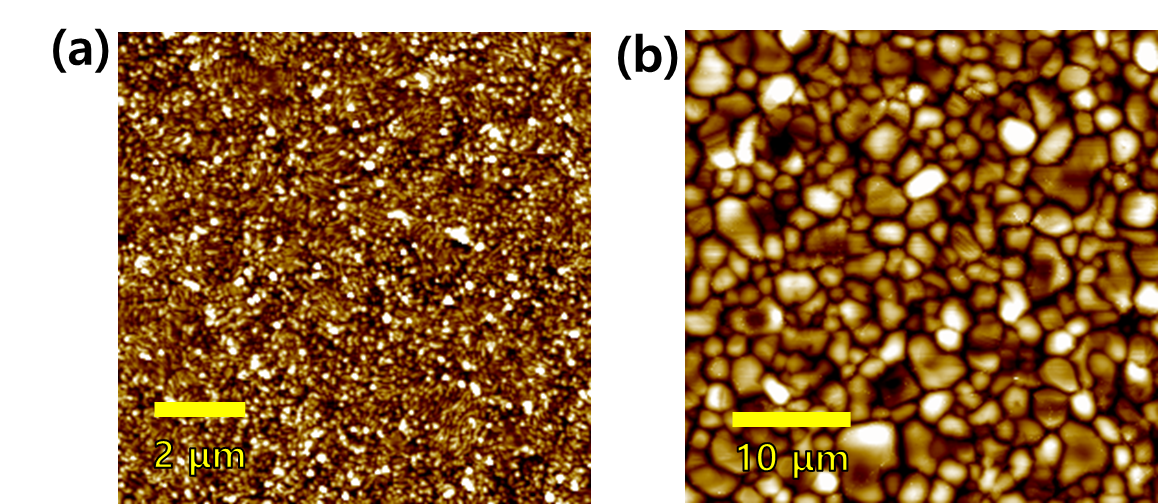


**Figure S4. AFM topography comparison of before and after thermal annealing.** AFM images on the surface of as deposited Cu(0.7μm)/Ni(0.3μm) substrate (**a**), and after CVD growth (**b**).


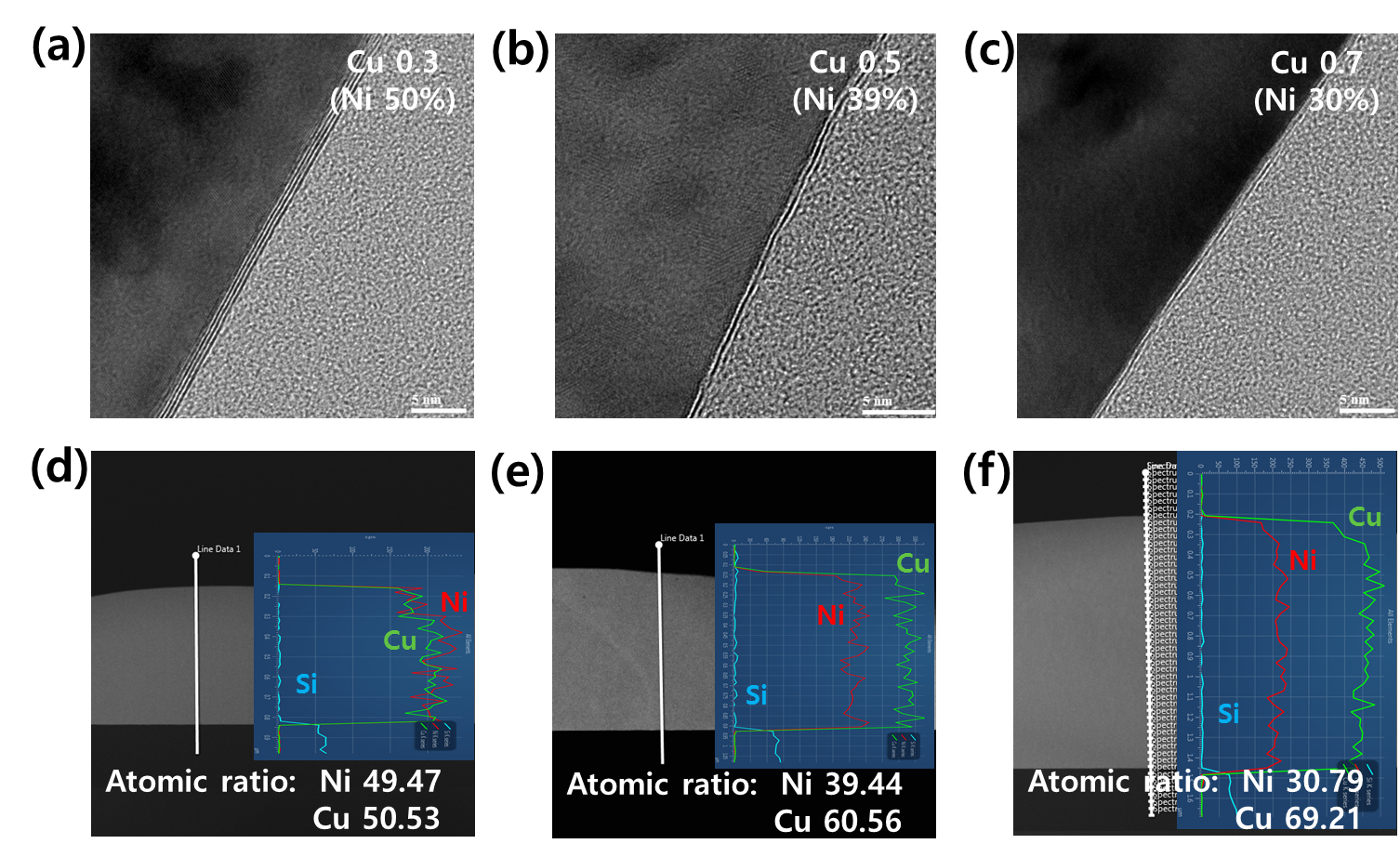


**Figure S5. Cross-sectional HRTEM and EDS elemental analysis of Cu0.3, Cu0.5 and Cu0.7.** **a-c,** Representative layers of graphene for each sample is shown. **d-f,** In-depth Cu-Ni atomic composite profile and their atomic ratio calculations.


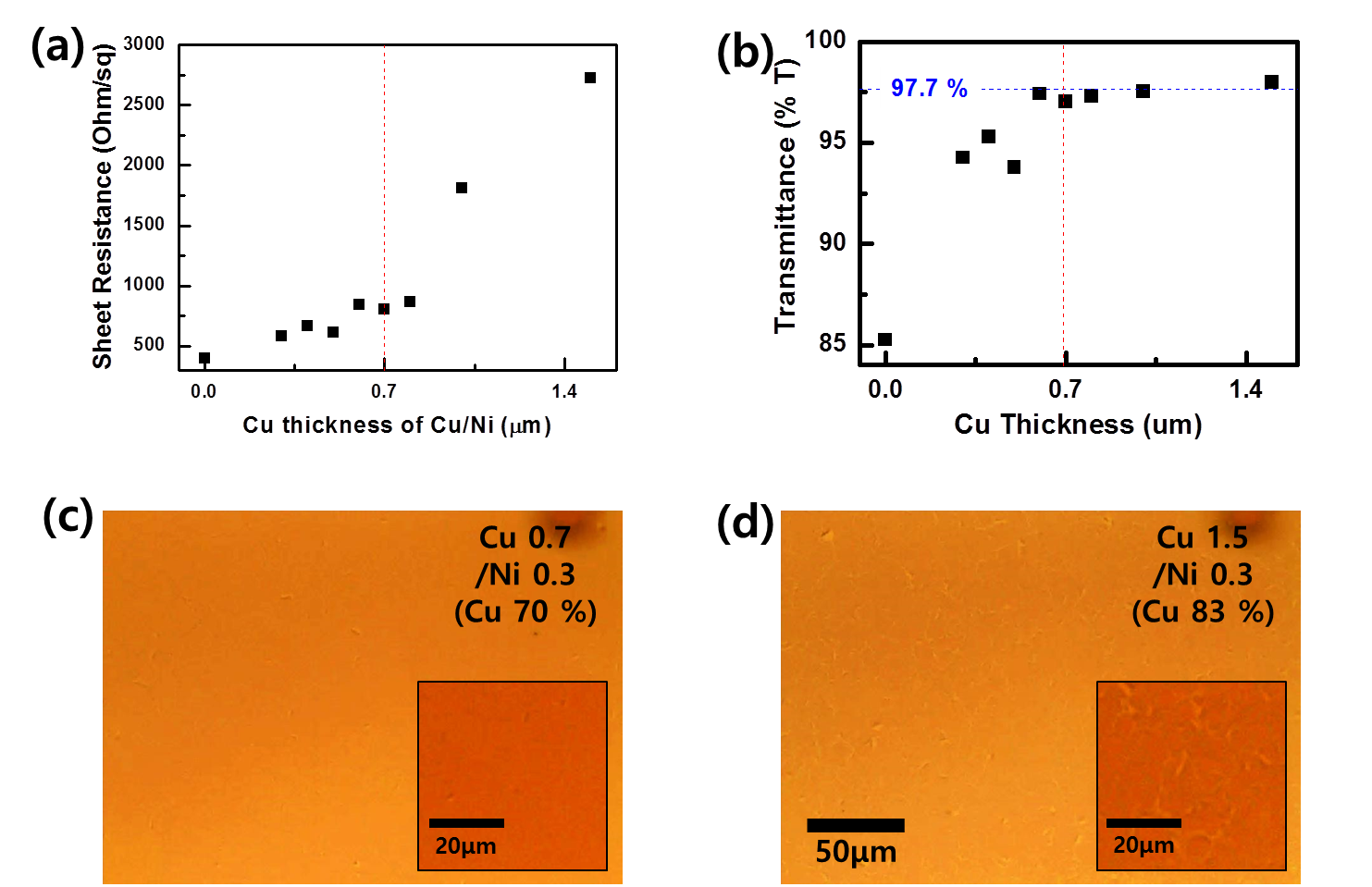


**Figure S6. Effects of cracks to the properties of SLG.** Cu thickness dependent Rs (**a**) and Tr (**b**) including the results of Cu0.8, Cu1.0, and Cu1.5. An increase of cracks are compared between optimal thickness of Cu0.7 (**c**) and over deposited Cu1.5 (**d**).


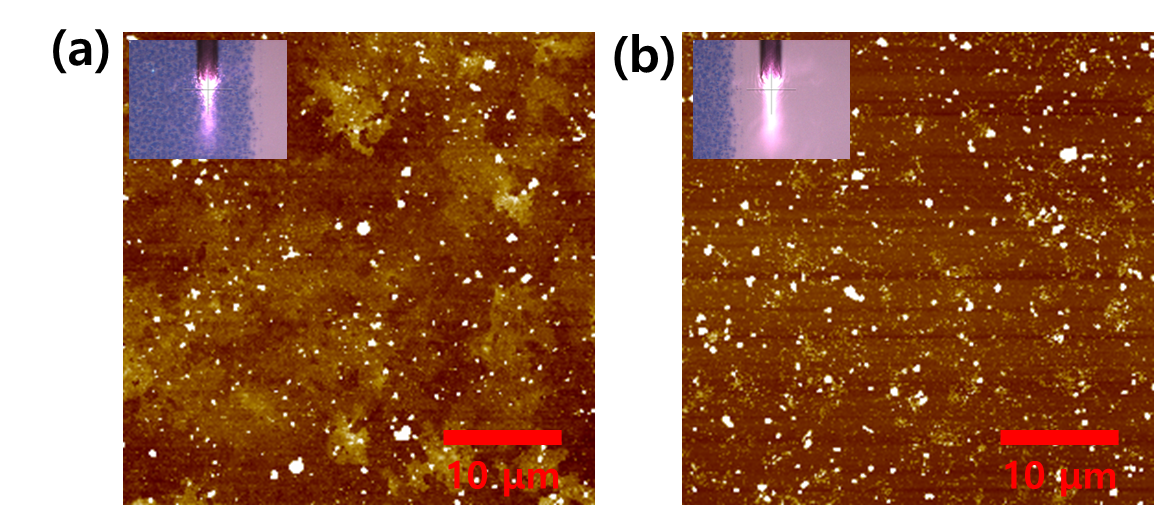


**Figure S7. AFM topographic images on MLG-SLG patterned sheet transferred onto SiO_2_ substrate.** 40 × 40 μm^2^ scan of AFM topography obtained on MLG (**a**), and SLG (**b**). Inset shows the cantilever position.


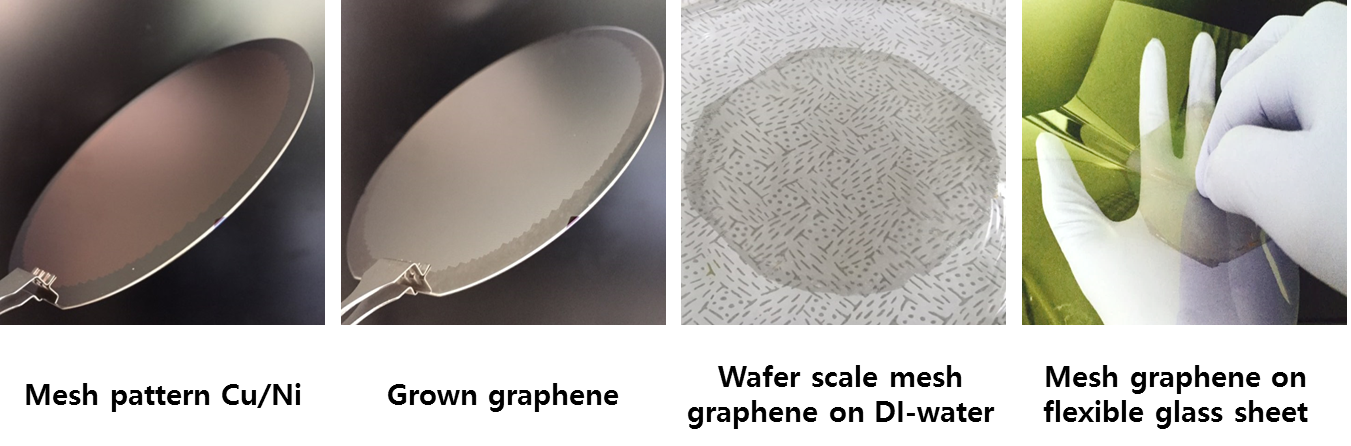


**Figure S8. Photographs of wafer-scale mesh pattern sheet growth and transfer to a flexible glass sheet.** A wafer-scale (4”) growth and transfer onto a flexible glass sheet (Schott, AF32R ECO Thin Glass, 50 μm thickness, 20 $\times$ 20 cm^2^ size) with whole mesh patterned graphene (square size: 200 $\times$ 200 μm^2^, line width: 100 μm), and bending test.


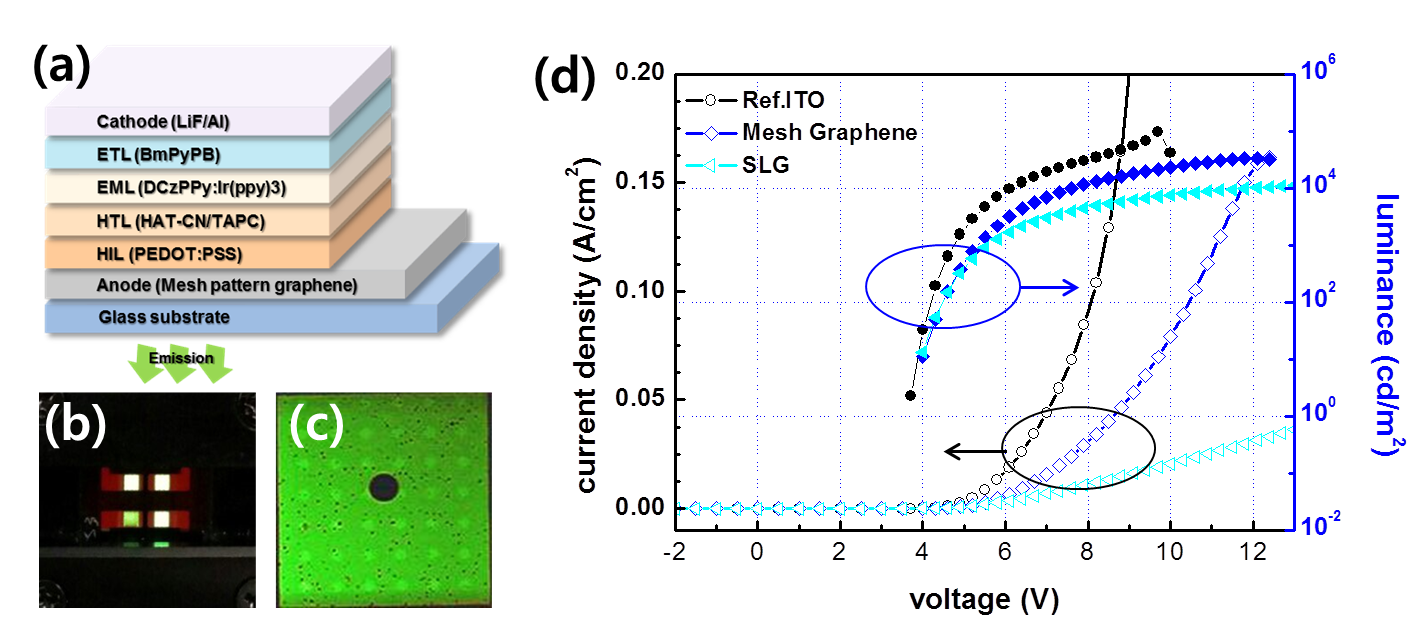


**Figure S9. Graphene based OLED device fabrication.** **a,** The device structure of graphene OLED. **b,** Image of light emitting with mesh pattern graphene OLED and pixel image of (**c**) mesh pattern graphene OLED. **d,** The current density-voltage-luminescence (JVL) characteristics with ITO, SLG, and mesh pattern graphene electrode.

**References**

S1. Li, X. *et* *al*. Large-area synthesis of high-quality and uniform graphene films on copper foils. *Science* **324**, 1312-1314 (2009).

S2. Bae, S. *et al*. Roll-to-roll production of 30-inch graphene films for transparent electrodes. *Nature Nanotech.* **5**, 574-578 (2010).

S3. Chen, S. Synthesis and characterization of large-area graphene and graphite films on commercial Cu-Ni alloy foils. *Nano* *Lett*. **11**, 3519-3525 (2011).
